# β2 Adrenoceptor Signaling Modulates Myoblast Differentiation via miR‐374b‐5p/GSK3β and miR‐326‐3p/Tnfrsf11a Axes

**DOI:** 10.1002/cbin.70214

**Published:** 2026-09-26

**Authors:** Tatiana E. Koike, Cesar S. Fuziwara, Audrei R. Santos, Fernanda V. B. Delpupo, Tábata L. Nascimento, Andrei Rozanski, Marcelo G. Pereira, Patrícia C. Brum, Edna T. Kimura, Thomas A. Rando, Elen H. Miyabara

**Affiliations:** ^1^ Institute of Biomedical Sciences University of São Paulo São Paulo São Paulo Brazil; ^2^ Department of Biochemistry Federal University of São Paulo São Paulo São Paulo Brazil; ^3^ School of Physical Education and Sport University of São Paulo São Paulo São Paulo Brazil; ^4^ Broad Stem Cell Research Center University of California Los Angeles California USA

**Keywords:** miR‐326‐3p, miR‐374b‐5p, myoblast, terminal differentiation, β2 adrenergic signaling

## Abstract

Myoblast differentiation is a crucial step of skeletal muscle regeneration and is impaired in the absence of the β2 adrenoceptor (ADRβ2). Since miR‐374b and miR‐326 regulate cell differentiation, this study investigated if ADRβ2 regulates myoblast differentiation via miR‐374b‐5p and miR‐326‐3p. Primary myoblasts from ADRβ2 knockout (β2KO) mice were cultured in proliferation and differentiation media. In silico, cellular and molecular analyses were performed to evaluate NF‐κB signaling activation and validate mRNAs as miR‐374b‐5p and miR‐326‐3p targets in β2KO differentiating myoblasts. Differentiating myoblasts from β2KO mice had decreased mRNA levels of *Nr4a1, Myog, Myh3, Cdkn1c,* and *Tnni2* and expression of miR‐374b‐5p and miR‐326‐3p. Overexpression of these miRs increased fusion index and gene expression of myogenic markers of these cells. In silico analyses indicated that GSK‐3β and receptor activator of NF‐kB (Tnfrsf11a) had potential binding sites for miR‐374b‐5p and miR‐326‐3p, respectively. The dual‐luciferase reporter assay showed that *GSK‐3β* and *Tnfrsf11a* were targets of miR‐374b‐5p and miR‐326‐3p, respectively. Regarding miR‐326‐3p, there was a reduction of NF‐κB signaling and *NF‐κB* mRNA levels in differentiating myoblasts from β2KO mice, and an upregulation of *Tnfrsf11a* and a downregulation of *Tnfrsf11*. In summary, ADRβ2 modulates myoblast differentiation via miR‐374b‐5p/*GSK‐3β* and miR‐326‐3p/*Tnfrsf11a* axes.

**Abbreviations**
Acta1: actin alfa 1
Adgrg1: adhesion G protein‐coupled receptor G1
ADRβ2: β2 adrenoceptor
AKT: protein kinase B
BP: biological process
cAMP: cyclic adenosine monophosphate
Cdkn1c: cyclin dependent kinase inhibitor 1 C
circ‐UQCRC2: circular RNA‐ubiquinol‐cytochrome C reductase core protein 2
CREB: cAMP response element‐binding protein
DEGs: differentially expressed genes
DESeq. 2: differential expression analysis for sequence count data
DM: differentiation medium
DMEM: Dulbecco's modified eagle medium
DMSO: dimethyl sulfoxide
dNTP: deoxynucleoside triphosphate
DTT: dithiothreitol
ECM: extracellular matrix
FDR: false discovery rate
FITC: fluorescein isothiocyanate
FK: forskolin
Fndc1: fibronectin type III domain‐containing protein 1
FoxO1: forkhead box O1
GAPDH: glyceraldehyde‐3‐phosphate dehydrogenase
GO: gene ontology
GSK‐3β: glycogen synthase kinase‐3 beta
HS: horse serum
LNA: locked nucleic acids
LPS: lipopolysaccharide
Mef2a: myocyte enhancer factor 2A
miRNAs: microRNAs
Mrc1: mannose receptor C‐type 1
Msr1: macrophage scavenger receptor 1
mTOR: mechanistic target of rapamycin
Myf5: myogenic factor 5
Myf6: myogenic factor 6
Myh3: myosin heavy chain ‐ embryonic
Mylpf: myosin light chain phosphorylatable, fast skeletal muscle
MyoD: myoblast determination protein 1
Myog: myogenin
NF‐κB: nuclear factor kappa B
Nr4a1: nuclear receptor subfamily 4 group A member 1
OPG: osteoprotegerin
Pax7: paired box 7
PBS: phosphate‐buffered saline
PDCD4: programmed cell death 4
PKA: protein kinase A
Plod1: procollagen‐lysine 2‐oxoglutarate 5‐dioxygenase 1
PM: proliferation medium
pRL‐CMV: co‐reporter vector containing the Renilla luciferase gene driven by the CMV promoter
RANK: receptor activator of nuclear factor κB
RANKL: receptor activator of nuclear factor κB ligand
RIN: RNA integrity number
RT‐qPCR: reverse transcription‐quantitative polymerase chain reaction
SD: standard deviation
Ssc5d: scavenger receptor cysteine‐rich family member with 5 domains
TA: tibialis anterior
Taf1: TATA‐box binding protein associated factor 1
Tnfrsf11: TNF receptor superfamily member 11
Tnfrsf11a: TNF receptor superfamily member 11a
Tnfrsf11b: TNF receptor superfamily member 11b
TNF‐α: tumor necrosis factor‐α
Tnni2: troponin I
UTR: untranslated region
WT: wild type
Xirp2: Xin actin‐binding repeat containing 2
β2KO: β2 adrenoceptor knockout

## 1 Introduction

The activity of muscle stem cells, known as satellite cells, enables the mammalian skeletal muscle to regenerate after damage. Under a mitotically quiescent state, these cells are located between the basal lamina and sarcolemma of myofibers and express the transcription factor Pax7 (Yin et al. 2013; Wosczyna and Rando 2018).

Muscle injury triggers an inflammatory response in the muscle, inducing the activation and proliferation of satellite cells, which promptly express Myf5, MyoD, and then myogenin, followed by their terminal differentiation into myofibers (Bentzinger et al. 2013; Dort et al. 2019). Most satellite cell‐derived myoblasts undergo myogenic differentiation (Yin et al. 2013; Bentzinger et al. 2013). However, a small percentage of proliferating satellite cells self‐renew to replenish the pool of quiescent satellite cells (Giordani et al. 2018; Rayagiri et al. 2018). Terminal myogenic differentiation is characterized by myoblast fusion, which is followed by myotube maturation, leading to myofiber repair (Yin et al. 2013; Wosczyna and Rando 2018). Finally, muscle regeneration involves angiogenesis, extracellular matrix (ECM) remodeling, and the recovery of muscle contractile function (Yin et al. 2013; Feige et al. 2018).

The function of satellite cells in skeletal muscle regeneration is impaired in the absence of the β2 adrenoceptor (ADRβ2) (Koike et al. 2022), suggesting that β2 adrenergic signaling is critical for satellite cell function. ADRβ2s represent the majority of β adrenoceptors (approximately 90%) in the skeletal muscle and are guanine nucleotide‐binding G protein‐coupled receptors, which bind catecholamines (Kim and Sainz 1992). The canonical β2 adrenergic pathway Gα‐adenylyl‐cyclase‐cAMP is implicated in skeletal muscle hypertrophy (Gonçalves et al. 2019), and β2 adrenergic stimulation triggers anabolic and anti‐catabolic effects in skeletal muscle via protein synthesis mediated by the AKT–mTOR–S6 kinase axis (Hagg et al. 2016). In addition, activated cAMP can interact with the protein kinase A (PKA), which phosphorylates calpastatin, resulting in the inhibition of the proteolytic activity of calpains (Lynch and Ryall 2008).

The absence of ADRβ2 impairs satellite cell self‐renewal and myoblast proliferation and differentiation (Koike et al. 2022). In addition, the dysregulation of Notch and Wnt/β‐catenin signaling impairs myoblast proliferation and differentiation, respectively (Koike et al. 2022).

MicroRNAs (miRNAs) are endogenous non‐coding RNAs that control gene expression by binding to the 3′‐UTR of target genes to inhibit translation or promote mRNA degradation (Singh et al. 2020). miRNAs affect myoblast proliferation and differentiation (Diniz and Wang 2016). Thus, we hypothesized that ADRβ2 controls myoblast differentiation via miRNAs.

Although miR‐374b and miR‐326 regulate cell differentiation (Ma et al. 2015; Jadideslam et al. 2018), it is unknown whether these miRNAs mediate the regulation of myoblast differentiation by ADRβ2. This study investigated the role of miR‐374b‐5p and miR‐326‐3p in ADRβ2‐mediated myoblast differentiation.

## 2 Materials and Methods

### 2.1 Animals

Friend virus B‐type wild‐type (WT) and ADRβ2 knockout (β2KO) mice (Chruscinski et al. 1999) (male, 2–3 months old) were utilized in this study. Animals were kept in an animal facility at the Department of Anatomy, Institute of Biomedical Sciences, University of São Paulo. All experiments were conducted according to the ethical principles and guidelines of the Brazilian Society of Animal Experimentation and were approved by the Animal Research Ethics Committee of the Institute of Biomedical Sciences of the University of São Paulo (Protocol No. 106/2017, 7549191022).

### 2.2 Muscle Injuries and Isolation of Primary Myoblasts

Animals were anesthetized, and the left tibialis anterior (TA) muscles were freeze‐injured, whereas the contralateral TA muscles were used as uninjured controls to evaluate the expression of miRs at 10 days post‐injury (dpi), a period characterized by myoblast differentiation and fusion with pre‐existing myofibers (Koike et al. 2022). Other mice were anesthetized, and their muscles from the hindlimbs were injured using a 30‐gauge needle (60 needle punctures), as described previously (Koike et al. 2022). After 3 days, injured muscles were removed and enzymatically digested with collagenase type II (500 units per mL; Invitrogen) in Ham's F10 medium containing 10% horse serum (HS) for 90 min; and with collagenase type II (100 units per mL) and dispase (2 units per mL; Invitrogen) for 30 min (Koike et al. 2022). The cell suspension was filtered with a 40‐μm cell strainer (BD Biosciences). Myoblasts were seeded in 12‐well plates coated with Matrigel (Sigma Aldrich) diluted in DMEM (1:5) and cultured in Ham's F10 medium containing 20% fetal bovine serum, 10% HS, basic fibroblast growth factor (2.5 ng/mL), and 1% penicillin/streptomycin for 24 h until 60%–80% confluence. Then, myoblasts were cultured in differentiation medium (DM) containing DMEM, 2% HS, and 1% penicillin/streptomycin for 24 or 48 h (Wen et al. 2012; Dort et al. 2019).

Incubation of myoblasts from WT mice with 20 μM ICI‐118, 551 (Sigma), an ADRβ2 antagonist, or its vehicle, 1 × PBS in DM was performed for 48 h (Wan et al. 2023). Myoblasts from β2KO mice were incubated with 40 μM forskolin (FK; Sigma), an adenylyl cyclase activator, or its vehicle dimethyl sulfoxide (DMSO; 1.3:400) in DM for 48 h (Frese et al. 2015). Incubation of myoblasts from WT mice with DMSO in DM for 48 h was also performed.

### 2.3 Immunofluorescence

After differentiation, myoblasts untransfected or transfected with 10 nM anti‐miRs were fixed in 4% paraformaldehyde for 30 min, permeabilized with 0.5% Triton X‐100, and blocked with 1% normal goat serum in PBS for 15 min. Cells were incubated with primary monoclonal antibody mouse anti‐desmin (1:50; Dako), primary polyclonal antibody rabbit anti‐desmin (1:100; Invitrogen), primary monoclonal antibody rabbit anti‐GSK‐3β (1:70; Cell Signaling), primary polyclonal antibody rabbit anti‐Tnfrsf11a (1:70; Bioss), or primary polyclonal antibody rabbit anti‐Myh3 (1:500; OriGene) overnight at 4°C. After washing in PBS, cells were incubated with the secondary antibody FITC‐conjugated donkey anti‐mouse (1:250; Jackson Immuno Research), or Rhodamine‐conjugated goat anti‐rabbit (1:250; Jackson Immuno Research). Cells were imaged using a fluorescence microscope (Observer D1, Zeiss). The percentage of the expression of GSK3β and Tnfrsf11a per cross‐sectional field (average of 3.5 and 4.1 random fields per biological replicate, respectively; each field = 199606 μm^2^) of differentiating myoblasts (*n* = 3 and 4 biological replicates, respectively) was quantified using ImageJ software.

### 2.4 Western Blotting

Western blot analysis was performed as described previously (Koike et al. 2022). Membranes were incubated with the primary monoclonal antibody rabbit anti‐GSK‐3β (1:1000; Cell Signaling), primary polyclonal antibody rabbit anti‐Tnfrsf11a (1:1000; Bioss), and mouse polyclonal anti‐glyceraldehyde‐3‐phosphate dehydrogenase (GAPDH; 1:1000; Cell Signaling). Immunoreactive bands were visualized using peroxidase‐conjugated AffiniPure goat anti‐rabbit IgG or peroxidase‐conjugated AffiniPure goat anti‐mouse IgG (Jackson ImmunoResearch Laboratories).

### 2.5 RNA Extraction and Reverse Transcription‐Quantitative Polymerase Chain Reaction (RT‐qPCR)

Total RNA was extracted using TRIzol reagent (Invitrogen). RNA (1 µg) was reverse transcribed into cDNA in a reaction mixture containing reverse transcriptase (200 U) (SuperScript II; Invitrogen), 1 μL of oligo‐dT (500 μg/mL, Invitrogen, USA), 5× first‐strand buffer, DTT (0.1 M), dNTP mix (10 mM), and ultrapure water (Invitrogen). PCR was performed in a 10 μL reaction volume containing cDNA, forward and reverse primers, Platinum SYBR Green qPCR SuperMix‐UDG (Invitrogen), and nuclease‐free water (Invitrogen). PCR was performed on a Corbett Rotor‐Gene 6000 thermal cycler (Qiagen). Cycling conditions consisted of 40 cycles of 95°C for 15 s and 60°C for 60 s. The primers were synthesized by Integrated DNA Technologies or Thermo Fisher Scientific (Supporting Table S1). PCR reactions were performed in duplicate. The expression level of each target gene was normalized to *Taf1* mRNA levels using the 2^−ΔΔCt^ method.

### 2.6 RNA‐Seq

RNA integrity number (RIN) and sequencing depth (million reads) of differentiating myoblasts from WT and β2KO groups are shown in Supporting Table S2. Sequencing reads were mapped to the mouse reference genome version GRCm39. Annotations and transcripts from mouse GENCODE vM29 were used. Reads were mapped and counted using STAR version 2.7.10a (Dobin et al. 2013). Differential gene expression was assessed using the Bioconductor package DESeq. 2 version 1.48.2 (Love et al. 2014).

Library size differences were accounted for by estimating size factors using the poscounts method, and dispersions were fitted using the glmGamPoi method (Ahlmann‐Eltze and Huber 2021) (version 1.20.0). Statistical testing was performed using the Wald test, and results extracted at a threshold of α = 0.05. Log2 fold changes were subsequently shrunk using the apeglm estimator (Zhu et al. 2019) (version 1.30.0). Gene Ontology (GO) enrichment analysis for Biological Process (BP) terms was performed separately for up‐ and down‐regulated DEGs using the enrichGO function from clusterProfiler (Xu et al. 2024) (version 4.16.0), applying the background universe of all genes passing DESeq. 2 independent filtering, with FDR correction and significance cutoffs of *p*. adjust < 0.05 and *q*‐value < 0.05. Redundant GO terms were reduced using simplify (semantic similarity cutoff = 0.7, retaining the term with the lowest adjusted p‐value per cluster). The RNA‐seq data used in this publication were deposited in the National Center for Biotechnology Information and are accessible through SRA data: PRJNA1532947.

### 2.7 miRNA Analysis

miRNAs that could bind to the *Adrb2* gene were identified using the TargetScan database (http://www.targetscan.org/vert_72/). Among them, we identified miR‐374 as a possible regulator of *Adrb2*. Since miR‐326 regulates differentiation of many cell types (Tang et al. 2009; Du et al. 2009; Choi et al. 2016; Po et al. 2017; Corral‐Fernández et al. 2017), we hypothesized that its expression could be altered in differentiating myoblasts lacking *Adrb2*. Myoblasts were isolated from WT and β2KO mice, as described previously (Koike et al. 2022). Myoblasts were seeded in 6‐well plates and cultured in proliferation medium (PM) for 24 h, and a fraction of these cells was incubated in DM for 24 or 48 h.

Total RNA was extracted from proliferating and differentiating myoblasts, and control and injured muscles at 10 dpi using TRIzol reagent (Invitrogen). RNA (100 ng) was reverse transcribed using the TaqMan MicroRNA Reverse Transcription kit (Thermo Fisher Scientific) and primers for snoRNA420, mmu‐miR‐326‐3p, or mmu‐miR‐374‐5p TaqMan MicroRNA Assays (Thermo Fisher Scientific), and the following cycling conditions: 16°C for 30 min, 42°C for 30 min, and 85°C for 5 min. qPCR was performed in a reaction mixture containing 1.2 μL of cDNA (100 ng/μL), 1 uL of TaqMan probes for each miRNATaqMan MicroRNA Assays (Thermo Fisher Scientific), 10 μL of TaqMan Universal PCR Master Mix (Thermo Fisher Scientific), and nuclease‐free water. Cycling conditions consisted of 40 cycles of 95°C for 15 s and 60°C for 30 s. PCR reactions were performed in duplicate.

miR‐374b was also identified by in silico analysis as a potential regulator of *Gsk3β*, whose expression was increased in differentiating myoblasts from β2KO mice (Koike et al. 2022). In addition, we performed in silico analysis of miR‐326 targets, and TNF receptor superfamily member 11a (*Tnfrsf11a*) was pointed as a potential target of this miRNA.

#### 2.7.1 Transfection With miRNA Mimics and Inhibitors

*miR‐374b‐5p* and *miR‐326‐3p* overexpression or inhibition were performed using mirVana miRNA mimics (Ambion, Austin, TX) to overexpress mmu‐miR‐374b‐5p or mmu‐*miR‐326‐3p*, or the mirVana miRNA inhibitor (Ambion) containing locked nucleic acids (LNA). Primary myoblasts (5.0 × 10^4^ cells) from WT or β2KO groups were seeded in 12‐well plates and cultured for 48 h. Cells were transfected with 10 nM miRNA mimics specific for miR‐374b‐5p and miR‐326‐3p or anti‐miR using Lipofectamine 2000 (Invitrogen) in Opti‐MEM according to the manufacturer's instructions. Total RNA was extracted using TRIzol, and mRNA levels and miRNA expression were quantified by qPCR as described previously. To examine whether overexpression of miR‐374b‐5p and miR‐326‐3p can rescue the differentiation defect of β2KO myoblasts, the fusion index and gene expression of myogenic markers of β2KO myoblasts untransfected or transfected with 10 nM miRNA mimics specific for miR‐374b‐5p and miR‐326‐3p, incubated in differentiation media for 48 h, were determined. Using a 40× objective and phase contrast, random fields were analyzed. Myotubes were defined as cells with three or more nuclei. The fusion index was determined as the percentage of nuclei in myotubes compared with the total number of nuclei in the field. Three different biological replicates were analyzed per group.

#### 2.7.2 Dual‐Luciferase Reporter Assay

##### 2.7.2.1 Validation of Gsk3β mRNA as a miR‐374b‐5p Target

miRNA target prediction analysis using the TargetScan and miRDB databases showed that *Gsk3b* had two predicted binding sites for miR‐374b‐5p (Supporting File 1). A segment of mouse *Gsk3β* 3′‐UTR containing the predicted miRNA binding site (nucleotides 2738‐2744) was cloned into the pmiRGlo luciferase reporter vector (Promega, Madison, WI, USA) in the sites for XhoI and XbaI that are located downstream of the firefly Luc gene to generate the pmiRGlo‐Gsk3β‐wt plasmid. pmiR‐Glo plasmid also contains the Renilla luciferase gene that is used as normalization control. Primers were designed to create the predicted binding site for miR‐374b‐5p, a restriction site for *Xho*I in the 5′ end, and a restriction site for *Xba*I in the 3′ end when annealed (Supporting Table S3). A mutated plasmid (pmiRGlo‐Gskβ‐mut) was constructed by annealing primers to contain mutations in this binding site (seed region) as described in Figure S1. The sequences of WT and mutated 3′‐UTRs cloned in the plasmids were confirmed by Sanger sequencing.

Primary myoblasts (5.0 × 10^4^ cells) were seeded in 24‐well plates in triplicate and transiently co‐transfected with 1 μg of pmiRGlo‐Gsk3β‐wt/pmiRGlo‐Gsk3β‐mut and 10 nM miR‐374b‐5p or 10 nM anti‐miR‐374b‐5p using Lipofectamine 2000. Cells were also transfected with a negative control (miR‐17‐5p) to determine the binding specificity of miR‐374b‐5p. Forty‐eight h after transfection, cells were lysed in 1× passive lysis buffer (Promega), and firefly and Renilla luciferase activity were measured sequentially using the Dual Luciferase Reporter Assay System (Promega) on a GloMax 20/20 Luminometer (Promega). Mean and SD were calculated by dividing the firefly luciferase values (target) by the Renilla luciferase values in the same sample. Comparisons were performed between experimental samples and controls (‐miR and miR‐17).

##### 2.7.2.2 Validation of Tnfrsf11a mRNA as a miR‐326‐3p Target

miRNA target prediction analysis using the TargetScan database showed that *Tnfrsf11a* had one potential binding site for miR‐326‐3p. A segment of the mouse *Tnfrsf11a* 3′‐UTR containing the predicted binding site (nucleotides 852‐859) was cloned into pmiRGlo (Promega, Madison, WI) in the sites for XhoI and XbaI that are located downstream of the Luc gene to generate the pmiRGlo‐*Tnfrsf11a*‐wt plasmid. The pmiR‐Glo plasmid also contains the Renilla luciferase gene that is used as a normalization control. Primers were designed to create the predicted binding site for miR326‐3p, a restriction site for *Xho*I in the 5′ end, and a restriction site for *Xba*I in the 3′ end when annealed (Supporting Table S3). A mutated plasmid (pmiRGlo‐*Tnfrsf11a*‐mut) was constructed by annealing primers to contain mutations in this binding site (seed region) (Figure S2). The sequences of WT and mutated 3′‐UTRs cloned in the plasmids were confirmed by Sanger sequencing. Primary myoblasts (5.0 × 10^4^ cells) were seeded in 24‐well plates and transiently transfected with 1 μg of pmiRGlo‐*Tnfrsf11a*‐wt/pmiR‐Glo‐*Tnfrsf11a*‐mut in the presence of miR*‐326b‐3p* mimic (10 or 30 nM) or anti‐miR‐326‐3p (10 or 30 nM) using Lipofectamine 2000. Cells were also transfected with a negative control (miR‐19b‐5p) to determine the binding specificity of miR‐326‐3p. Forty‐eight h after transfection, cells were lysed in 1× passive lysis buffer (Promega), and firefly and Renilla luciferase activity was measured as described before. Mean and SD were calculated by dividing the firefly luciferase values (target) by the Renilla luciferase values in the same sample. The negative control group (miR‐19) was used as the reference and normalized to 1; all other group values were expressed relative to this control.

### 2.8 NF‐κB Signaling Activation Assay in Myoblasts in DM

We used a consolidated NF‐κB reporter plasmid pGL4.32[luc2P/NF‐kB‐RE/Hygro] that aligns five copies of NF‐κB binding sites (Promega) to control firefly luciferase expression.

Myoblasts were isolated from three to four animals from each group and seeded in gelatin‐coated 24‐well plates (50,000 cells/well). After 24 h, the culture medium was exchanged for PM, and cells were cultured for another 24 h. Cells were cotransfected with 300 ng of pGL4.32[luc2P/NF‐kB‐RE/Hygro] (NF‐κB reporter) or a control vector and 30 ng of pRL‐CMV (containing the Renilla luciferase gene driven by the CMV promoter) using Lipofectamine 2000 (1 µg DNA: 2 µL lipofectamine) in Opti‐MEM medium. After 4 h of transfection, the medium was exchanged for DM. Forty‐eight h after transfection, cells were lysed in 1× passive lysis buffer (Promega). Luminescence was quantified using the Dual Luciferase Reporter as described before. Mean and SD were calculated by dividing firefly luciferase values (target) by the Renilla luciferase values in the same sample. WT group values were normalized to 1, and β2KO values were compared accordingly.

### 2.9 Statistical Analysis

Statistical analyses were performed using GraphPad Prism version 8. Data were expressed as the mean ± standard deviation or mean ± standard error of the mean, and the significance of differences between means was determined using an unpaired t‐test (pairwise comparisons) or one‐way analysis of variance followed by Tukey's post hoc test (multiple comparisons). A *p*‐value of less than 0.05 was considered statistically significant.

## 3 Results

### 3.1 β2KO Upregulates Genes Associated With ECM and Inflammation, and Downregulates Genes Related to Myoblast Differentiation and Muscle Structure and Contractile Function

**Figure 1:**
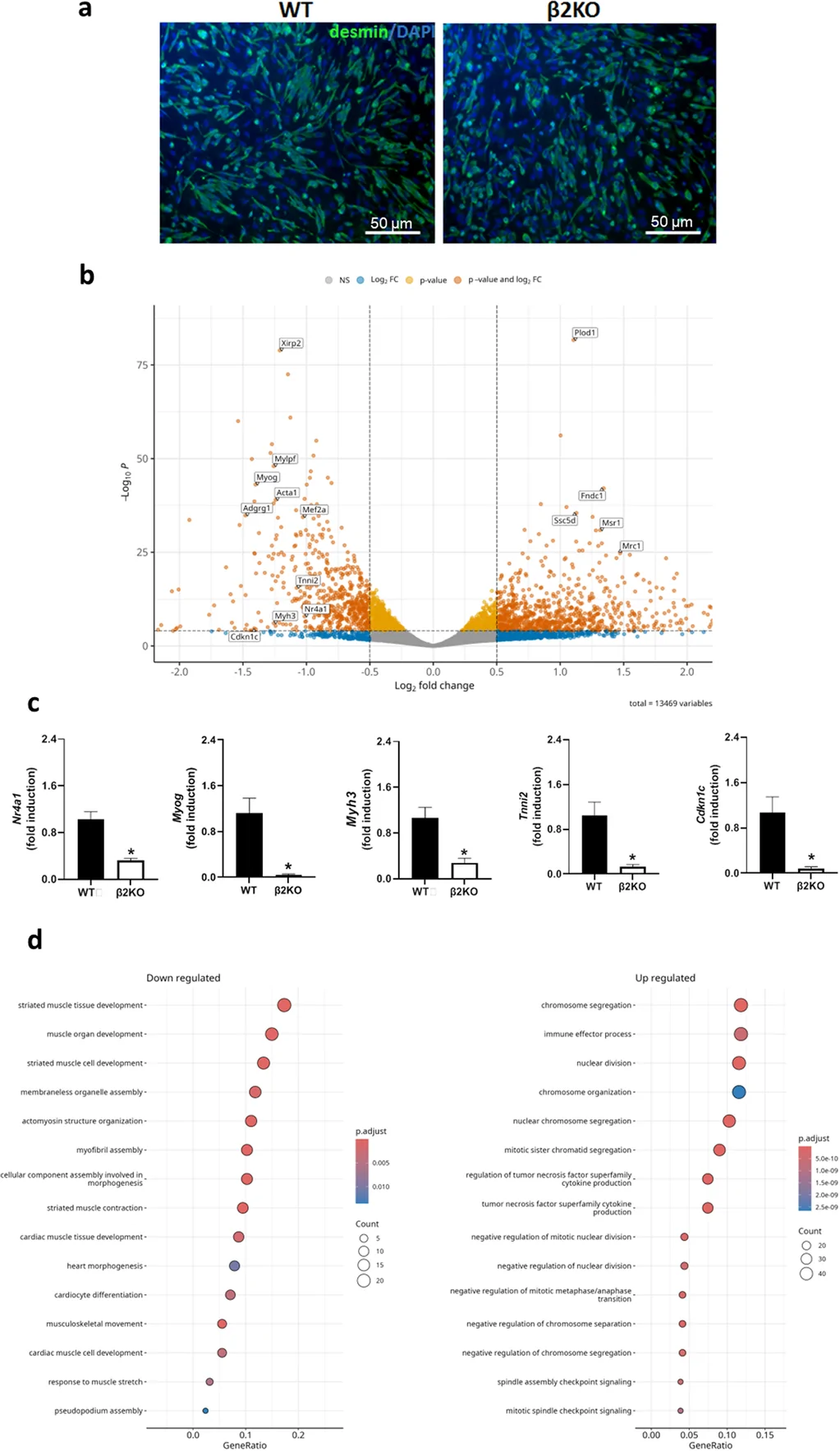
(a) Representative immunostaining of desmin‐positive myoblasts from β2KO and WT groups cultured in differentiation medium (DM) for 48 h. Blue: DAPI (nuclear staining); Green: desmin. Bar: 50 μm. (b) Volcano plot depicting results of DESeq. 2 differential expression analysis comparing β2KO and WT differentiating myoblasts (*n* = 3 biological replicates). The *x*‐axis shows the shrunken log_2_ fold change (lfcShrink, apeglm); the *y*‐axis shows the −log_10_ raw p‐value. Genes *Myog, Tnni2, Nr4a1, Myh3*, and *Cdkn1c* qPCR validation are shown in Figure 1c. (c) mRNA levels of *Myog* (myogenin; *n* = 4 biological replicates), *Tnni2* (Troponin I; *n* = 3–6 biological replicates), *Nr4a1* (nuclear receptor subfamily 4, group A, member *1*; *n* = 4 biological replicates*), Myh3* (myosin heavy chain – embryonic; *n* = 4–5 biological replicates), and *Cdkn1c (*cyclin dependent kinase inhibitor 1 C; *n* = 3–6 biological replicates*)* in myoblasts cultured in DM for 48 h (validation of RNAseq data). Groups: WT; β2KO. Data are expressed as mean and SEM. (Student t‐test) *p ≤ 0.05 versus WT group. (d) GO BP enrichment dotplots for up‐ and down‐regulated genes (padj < 0.01, log2FC > 1.0). Top 15 terms shown after semantic simplification (cutoff = 0.7).

First, transcriptomic profile was assessed to detect overall alterations of gene expression in myoblast differentiation in the absence of ADRβ2 (Figure 1a). Differential gene expression analysis revealed upregulation of genes involved in the regulation of ECM (such as *Plod1, Fndc1*) and immune effector process (such as *Ssc5d, Msr1, Mrc1*) (Figure 1b,d), which is in line with previous studies that showed the absence of ADRβ2 during skeletal muscle regeneration increases ECM remodeling (Silva et al. 2016) and inflammatory infiltration (Silva et al. 2014).

In contrast, pronounced downregulation was observed for genes associated with striated muscle development (such as *Mylpf* and *Mef2a*), structure (such as *Xirp2* and *Adgrg1*), and function (such as *Acta1*) (Figure 1b), in agreement with Gene Ontology enrichment analysis (Figure 1d).

PCR analysis demonstrated that β2KO downregulated *Myog* (myogenin) and *Myh3* (myosin heavy chain ‐ embryonic), markers of early and late myoblast differentiation (myoblast fusion) (Zammit et al. 2006); *Cdkn1c* gene (cyclin dependent kinase inhibitor 1 C), which helps drive terminal muscle differentiation (Osborn et al. 2011); *Tnni2* gene (troponin I), which is involved in muscle contraction (Marston and Zamora 2020); and *Nr4a1* (nuclear receptor subfamily 4, group A, member 1), a cAMP response element‐binding protein (CREB) target gene (Kanzleiter et al. 2009; Maruoka et al. 2020) (Figure 1c) in accordance with RNAseq results. These results are in accordance to a previous study, which demonstrated that the lack of the ADRβ2 impairs myoblast differentiation and affects downstream signaling of the canonical β2‐adrenergic pathway Gα‐adenylyl‐cyclase‐cAMP in myoblasts (Koike et al. 2022).

### 3.2 ADRβ2 Regulates Myoblast Differentiation Through miR‐374b‐5p‐GSK3β Signaling

**Figure 2:**
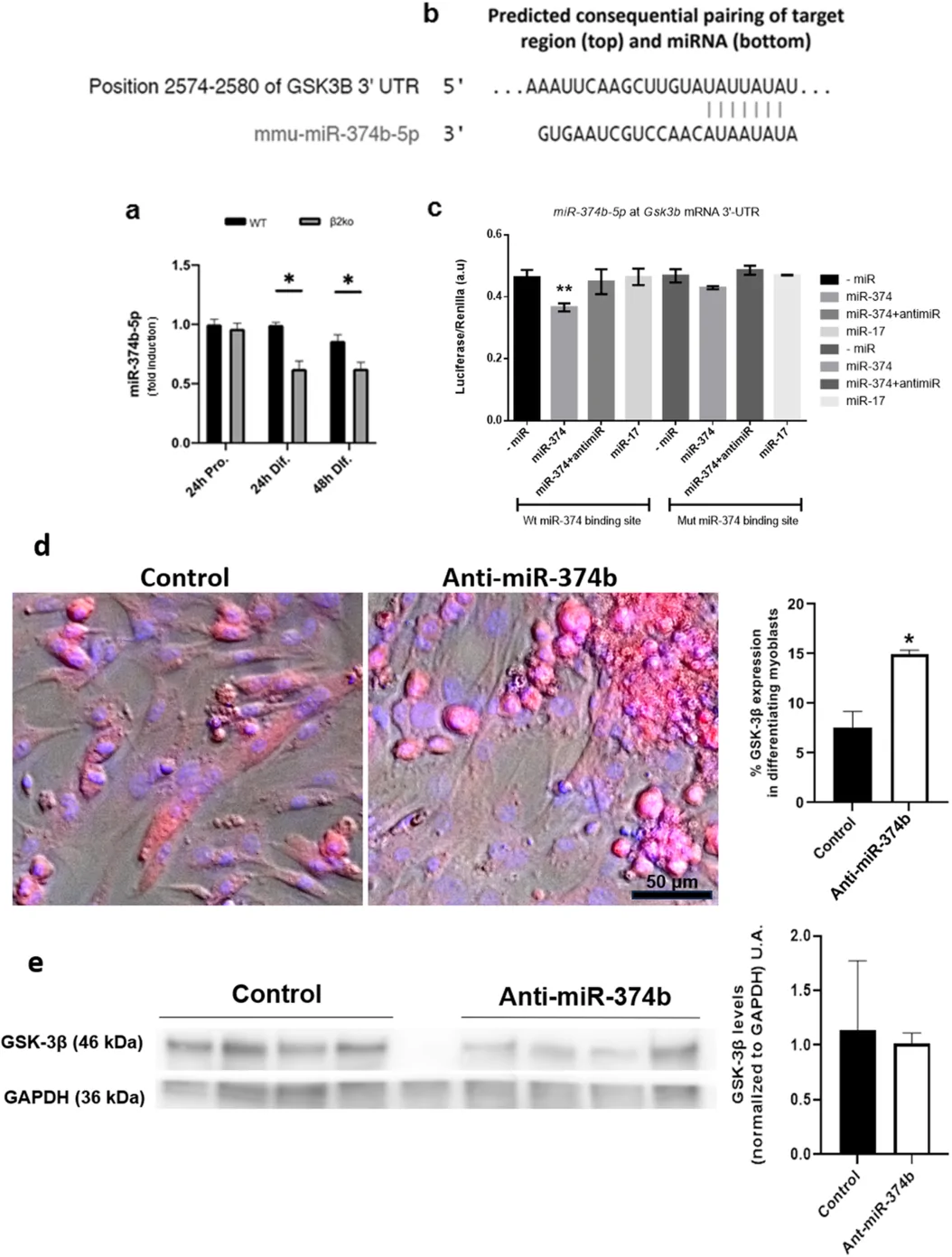
(a) Expression of miR‐374b‐5p in myoblasts from β2kO cultured in growth medium (GM) for 24 h and in DM for 24 and 48 h. Data are expressed as mean and SEM (*n*: 4‐5 biological replicates). ANOVA followed by Tukey's test. *p ≤ 0.05 versus respective WT. (b) Predicted binding sites of miR‐374b‐5p in the 3′‐UTR mRNA of *Gsk3β* (TargetScanHuman 7.0 predicted targeting of mouse *Gsk3β)*. (c) Validation of *GSK3β* mRNA as a miR‐374b‐5p target in primary myoblast cells by luciferase assay. The plasmid pmiR‐Glo contains the wild‐type predicted binding site for miR‐374b‐5p in 3′‐UTR, or the mutated site, and modulation of miR‐374b‐5p expression was performed with 10 nM miRNA mimics or antimiRs. miR‐17 mimic was used as a non‐target control. Firefly luciferase activity was normalized to Renilla luciferase activity. ANOVA followed by Tukey's test. **p ≤ 0.01 versus –miR or miR‐17. a.u.: arbitrary units. Mean ± SD of three different biological replicates per group. Representative results of two independent experiments performed in triplicate. (d) Representative immunostaining of GSK3β in differentiating myoblasts from the WT group transfected with 10 nM control anti‐miR or anti‐miR‐374b‐5p cultured in differentiation medium (DM) for 48 h. Blue: DAPI (nuclear staining) and Red: GSK3β merged with phase contrast field. Bar: 50 μm. Expression of GSK3β in differentiating myoblasts from the WT group transfected with control anti‐miR or anti‐miR‐374b‐5p cultured in differentiation medium (DM) for 48 h. Percentage of the expression of GSK3β per cross‐sectional field (average of 3.5 random fields per biological replicate; each field = 199606 μm^2^) of differentiating myoblasts (*n* = 3 biological replicates). Data are expressed as mean and SEM. Groups: Control; Anti‐miR‐374b. Student *t*‐test, *p ≤ 0.05 versus Control group. (e) Representative western blot and densitometric analysis of GSK3β in differentiating myoblasts from the WT group transfected with 10 nM control anti‐miR or anti‐miR‐374b‐5p cultured in differentiation medium (DM) for 48 h (*n* = 4 biological replicates per group). GAPDH was used as a standard. Data are expressed as mean and SEM. Student *t*‐test. Groups: Control; Anti‐miR‐374b.

Our previous study showed that β2KO increased the expression of *GSK3β* in differentiating myoblasts (Koike et al. 2022). Since miRNA expression is modulated during myoblast differentiation (Diniz and Wang 2016), we evaluated changes in miRNA expression in differentiating myoblasts lacking ADRβ*2*. The expression of miR‐374 decreased significantly in myoblasts from β2KO mice at 24 and 48 h of differentiation (Figure 2a). In addition, there was an increase in the expression of miR‐374 in uninjured TA muscles from β2KO mice, which was avoided on those injured TA muscles at 10 dpi from β2KO mice (Figure S3), a period of myoblast differentiation and fusion with regenerating myofibers (Koike et al. 2022). Target prediction analysis using the TargetScan database showed that *Gsk3β* 3′ UTR was a potential target of miR‐374b‐5p (Figure 2b).

To validate miR‐374b interaction with *Gsk3β* 3′ UTR, we transfected the pmiR‐Glo‐Gsk3β‐wt plasmid that contains the wt binding site for miR‐374b into myoblasts. In the presence of miR‐374b mimic, the relative luciferase activity was reduced in 22%, while the antimiR‐374b reverted this effect. Moreover, the transfection of the mutated plasmid abolished the mimic effect (Figure 2c). These results indicate that miR‐374b‐5p interacted with the WT 3′‐UTR of *Gsk3β*, and a 3′‐UTR mutation reduced this effect (Figure 2c). In addition, differentiating myoblasts transfected with anti‐miR‐374b‐5p for 48 h showed increased GSK3β expression via immunofluorescence (Figure 2d), whereas expression remained unaltered in Western blot analysis (Figure 2e). This demonstrates that, unlike Western blot analysis of whole‐cell lysates, immunofluorescence allows for a more sensitive detection of GSK3β expression in differentiating myoblasts. These findings demonstrate that miR‐374b‐5p targets *Gsk3β* mRNA in myoblasts.

### 3.3 ADRβ2 Regulates Myoblast Differentiation Through miR‐326‐3p‐Tnfrsf11a Signaling

**Figure 3:**
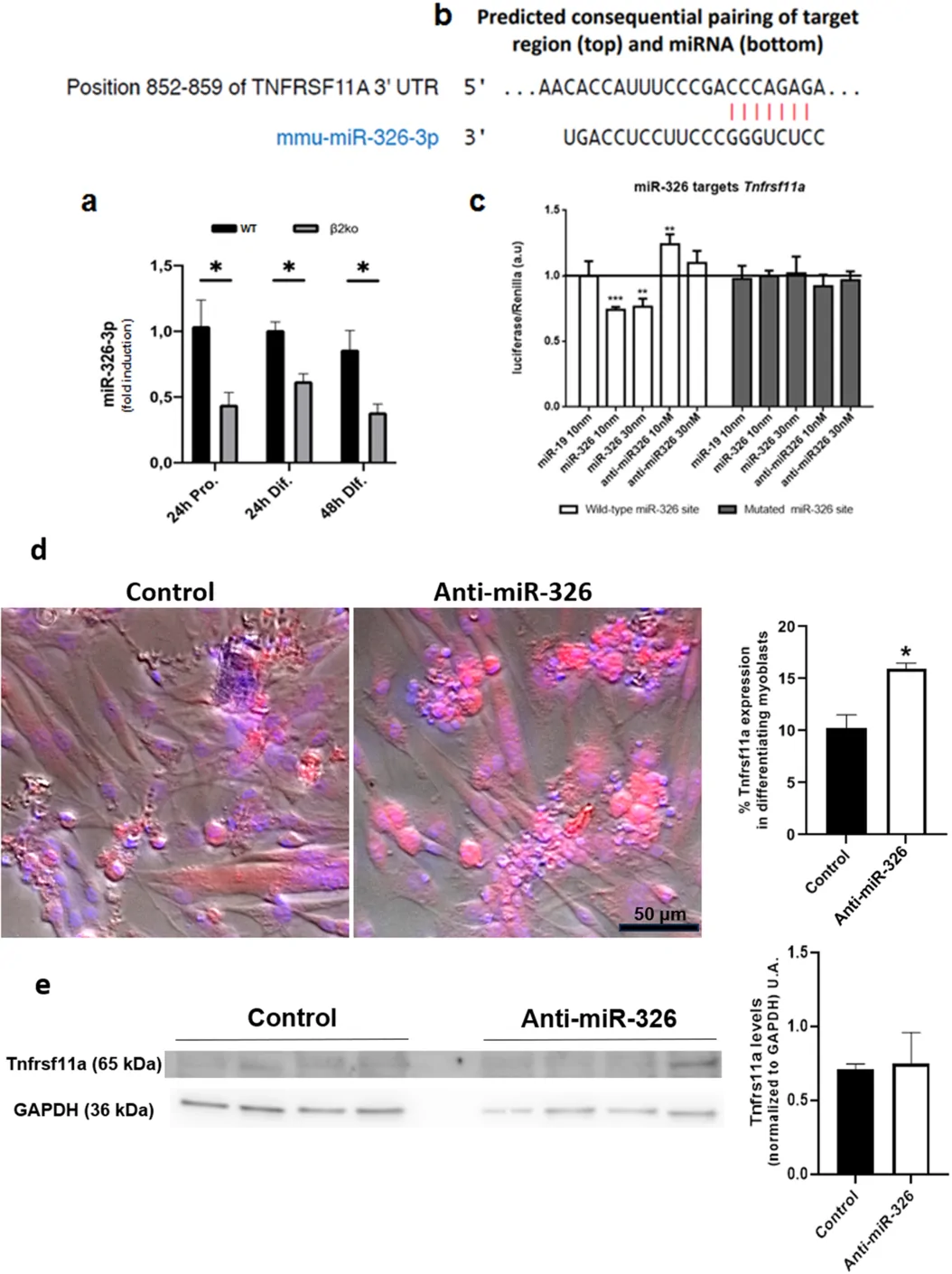
(a) Expression of miR‐326‐3p in myoblasts from β2KO cultured in growth medium (GM) for 24 h and in DM for 24 and 48 h. Data are expressed as mean and SEM (*n*: 3‐6 biological replicates). ANOVA followed by Tukey's test. *p ≤ 0.05 versus respective WT. (b) Predicted binding sites of miR‐326‐3p in the 3′‐UTR mRNA of *Tnfrsf11a* (TargetScanHuman 7.0 predicted targeting of mouse Tnfrsf11a). (c) Validation of *Tnfrsf11a* mRNA as a miR‐326‐3p target in myoblasts by luciferase assay. The plasmid pmiR‐Glo wt contains the wild‐type predicted binding site for miR‐326‐3p in 3′‐UTR, and the modulation of miR‐326‐3p expression was performed with 10 nM miRNA mimics or antimiRs. miR‐19 mimic was used as a non‐target control. Firefly luciferase activity was normalized to Renilla luciferase activity. ANOVA followed by Tukey's test. **p ≤ 0.01 versus miR‐19, ***p ≤ 0.001 versus miR‐19. a.u.: arbitrary units. Mean ± SD of four different biological replicates per group. (d) Representative immunostaining of Tnfrsf11a in differentiating myoblasts from the WT group transfected with control anti‐miR or anti‐miR‐326‐3p cultured in differentiation medium (DM) for 48 h. Blue: DAPI (nuclear staining) and Red: Tnfrsf11a merged with phase contrast field. Bar: 50 μm. Expression of Tnfrsf11a in differentiating myoblasts from the WT group transfected with 10 nM control anti‐miR or anti‐miR‐326‐3p cultured in differentiation medium (DM) for 48 h. Percentage of the expression of Tnfrsf11a per cross‐sectional field (average of 4.1 random fields per biological replicate; each field = 199606 μm^2^) of differentiating myoblasts (*n* = 4 biological replicates). Data are expressed as mean and SEM. Groups: Control; Anti‐miR‐326. Student *t*‐test, *p ≤ 0.05 versus Control group. (e) Representative western blot and densitometric analysis of Tnfrsf11a in differentiating myoblasts from the WT group transfected with 10 nM control anti‐miR or anti‐miR‐326‐3p cultured in differentiation medium (DM) for 48 h (*n* = 4 biological replicates per group). GAPDH was used as a standard. Data are expressed as mean and SEM. Student *t*‐test. Groups: Control; Anti‐miR‐326‐3p.

Considering that miR‐326‐3p regulates the differentiation of neural cells (Choi et al. 2016; Po et al. 2017), adipocytes (Tang et al. 2009), and lymphocytes (Du et al. 2009; Corral‐Fernández et al. 2017), we investigated whether β2KO downregulated miR‐326‐3p in myoblasts cultured in PM and DM. Indeed, the levels of miR‐326‐3p were reduced in myoblasts from the β2KO under both proliferation and differentiation conditions compared to the wild‐type (Figure 3a), as well as in TA muscles from β2KO mice at 10 dpi compared with those from WT mice (Figure S3). Target prediction analysis using the TargetScan database showed that miR‐326‐3p could bind to *Tnfrsf11a* (Figure 3b). RANKL (Tnfsf11) binds to its receptor Tnfrsf11a and activates NF‐κB signaling (Bonnet et al. 2019; Marcadet et al. 2022). We performed dual‐luciferase reporter assays to investigate the binding of miR‐326‐3p to the 3′‐UTR of *Tnfrsf11a* (Figure 3c). miR‐326‐3p mimic significantly decreased relative luciferase activity in cells transfected with pmiR‐Glo‐ Tnfrsf11a‐wt, and anti‐miR reversed this effect. In turn, miR‐326‐3p mimic did not affect luciferase activity in cells transfected with pmiR‐Glo‐Tnfrsf11a‐mut (Figure 3c). In addition, differentiating myoblasts transfected with anti‐miR‐326‐3p for 48 h showed increased Tnfrsf11a expression via immunofluorescence (Figure 3d), whereas expression remained unchanged in Western blot analysis (Figure 3e). This indicates that immunofluorescence analysis detected Tnfrsf11a expression in differentiating myoblasts with higher sensitivity than Western blot analysis of total protein extracts. These findings indicate that miR‐326‐3p targets *Tnfrsf11a* in myoblasts.

**Figure 4:**
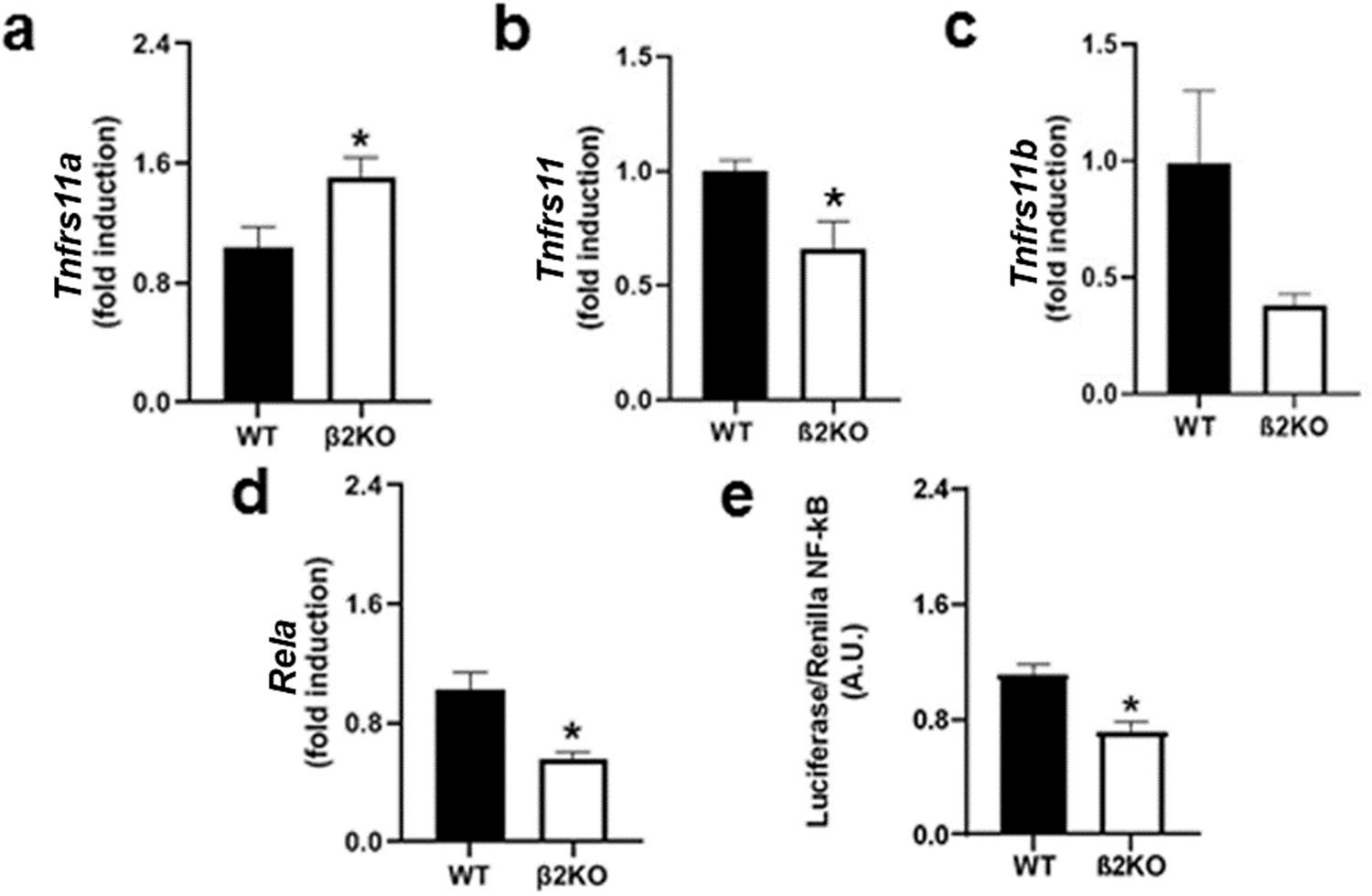
(a–d) mRNA levels of *Tnfrsf11a* (Rank), *Tnfsf11* (Rankl), *Tnfrsf11b* (OPG), and *Rela* (NF‐kB p65), respectively, in myoblasts cultured in DM for 48 h. Mean ± SEM; *n* = 4–8. (Student *t*‐test) *p ≤ 0.05 versus WT. (e) Deletion of β2 adrenoceptor in myoblasts cultured in DM for 48 h suppressed NF‐kB activation, evidenced by decreased luciferase activity of an NF‐kB luciferase plasmid containing five copies of an NF‐κB response element (NF‐κB‐RE). Luciferase activity of the NFkB reporter plasmid was normalized to the renilla luciferase expression of the pRL‐CMV vector. Mean ± SEM of 3–4 different biological replicates per group analyzed on two different days. (Student *t*‐test) *p ≤ 0.05 versus WT. A.U.: arbitrary units.

Gene expression analysis showed that β2KO led to the upregulation of *Tnfrsf11a*, in an inverse correlation with miR‐326 levels, downregulation of *Tnfrsf11* and *Rela* (which encodes NF‐κB p65), and a decrease in NF‐κB activity in differentiating myoblasts (Figure 4). In turn, β2KO did not affect the expression of *Tnfrsf11b* (which encodes osteoprotegerin [OPG]) (Figure 4). These results show that β2KO alters the expression of genes that encode components of the RANK‐RANKL‐OPG signaling during myoblast differentiation.

### 3.4 Canonical β2 Adrenergic Signaling Affects the Expression of miR‐374b‐5p and miR‐326‐3p During Myoblast Differentiation

To investigate the impact of the canonical β2 adrenergic signaling Gα‐adenylyl‐cyclase‐cAMP on differentiating myoblasts from WT mice, these cells were incubated with ICI, a beta 2 adrenergic antagonist (Wan et al. 2023). In addition, it was examined whether the incubation with the adenylyl cyclase activator FK can rescue the differentiation defect of β2KO myoblasts. Thus, fusion index and the expression of miRNA 326‐3p and miRNA 374b‐5p were determined from these myoblasts, incubated with FK or the vehicle DMSO in DM for 48 h, in comparison with those from myoblasts of WT mice incubated with the vehicle DMSO. The incubation of differentiating myoblasts with ICI for 48 h strongly decreased the fusion index (77% decrease) and reduced the expression of miR‐326, whereas there was a trend of decrease in the expression of miR‐374b (Figure S4). The exposure of differentiating β2KO myoblasts with FK for 48 h attenuated the decline in fusion index from these cells compared to WT myoblasts (34% decrease in β2KO FK and 66% decrease in β2KO Control *vs.* WT Control, Figure S4), and rescued of the expression of both miR‐374b‐5p and miR‐326‐3p (Figure S4). In addition, the expression of the cAMP response element‐binding protein (CREB) target gene *Nr4a1* was decreased at 48 h of differentiation of β2KO myoblasts (Figure 1c), corroborating that ADRβ2 knockout in myoblasts affects the downstream signaling of the canonical β2‐adrenergic pathway Gα‐adenylyl‐cyclase‐cAMP. Collectively, these results suggest that the canonical β2‐adrenergic signaling modulates myoblast differentiation via miR‐374b and miR‐326.

### 3.5 Overexpression of miR‐374b‐5p and miR‐326‐3p Rescues the Differentiation Defect of β2KO Myoblasts

**Figure 5:**
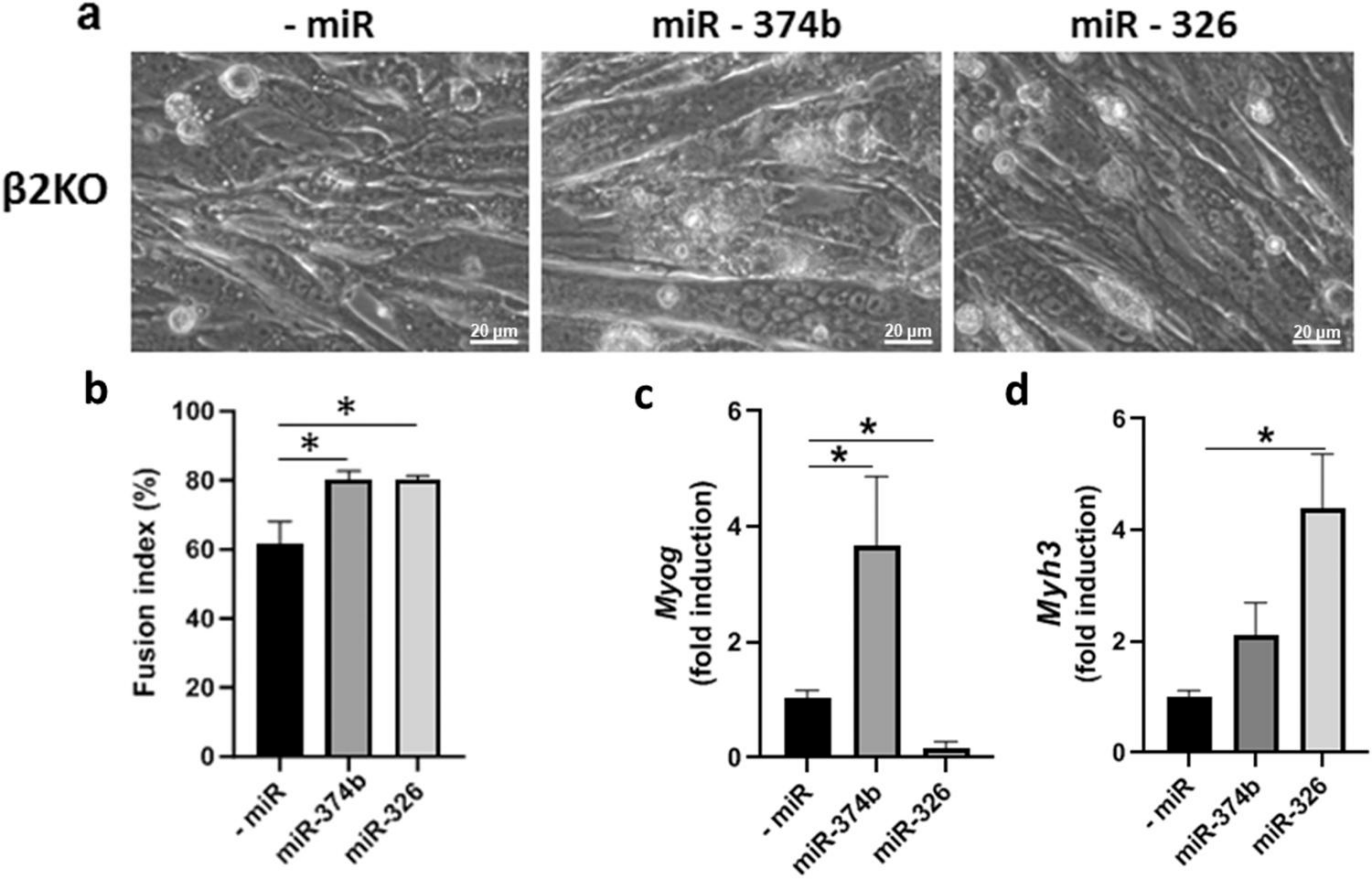
(a) Representative photomicrographs of β2KO myoblasts, untransfected (‐ miR) and transfected with 10 nM miRNA mimics specific for miR‐374b‐5p and miR‐326‐3p, in differentiation medium (DM) for 48 h (phase contrast fields). Bar: 20 μm. (b) Fusion index (%) of myoblasts incubated in differentiation media for 48 h. Groups: untransfected (‐ miR) and transfected with miR‐374 and miR‐326. Data are expressed as mean ± S.D.; *p ≤ 0.05 versus the ‐ miR group (analysis of variance followed by Tukey's test for multiple comparisons), *n* = 3 for each group ANOVA followed by Tukey's test. Mean ± SD of three different biological replicates per group. mRNA levels of (c) *Myog* (myogenin; *n* = 4 biological replicates), and (d) *Myh3* (myosin heavy chain – embryonic; *n* = 4–5 biological replicates) in myoblasts incubated in differentiation media for 48 h. Groups: untransfected (‐ miR) and transfected with miR‐374 and miR‐326. Mean ± SEM of 3–4 different biological replicates per group.*p ≤ 0.05 versus the ‐ miR group, ANOVA followed by Tukey's test.

To examine whether overexpression of miR‐374b and miR‐326 can rescue the differentiation defect of β2KO myoblasts, the fusion index was determined from these myoblasts, incubated in DM for 48 h, untransfected or transfected with miRNA mimics specific for miR‐374b‐5p and miR‐326‐3p. In fact, the fusion index of β2KO myoblasts transfected with miRNA mimics specific for miR‐374b‐5p and miR‐326‐3p increased 31% compared to untransfected β2KO myoblasts (Figure 5a,b). In addition, the expression of myogenic genes *Myog* and *Myh3* was increased in β2KO myoblasts transfected with miRNA mimics specific for miR‐374b‐5p and miR‐326‐3p, respectively (Figure 5c,d).

## 4 Discussion

The β2 adrenergic signaling regulates myoblast differentiation through the β2 adrenoceptor (Koike et al. 2022). In addition, miR‐374b and miR‐326 control cell differentiation (Ma et al. 2015; Jadideslam et al. 2018); however, it is still unknown whether myoblast differentiation controlled by ADRβ2 is mediated by these miRs. The present results showed that ADRβ2 modulates myoblast differentiation via miR‐374b‐5p/*GSK3β* and miR‐326‐3p/*Tnfrsf11a* axes, which have an *in vivo* impact since the absence of this receptor differentially affected the expression of miR‐374b and miR‐326 in uninjured and regenerating muscles, respectively.

Our RNA‐seq data analysis showed that β2KO in differentiating myoblasts upregulated genes related to ECM and inflammation. Indeed, previous studies reported that the lack of ADRβ2 during skeletal muscle regeneration favors ECM remodeling (Silva et al. 2016) and inflammatory infiltration (Silva et al. 2014). In addition, we found that β2KO in differentiating myoblasts downregulated genes associated with myoblast differentiation and muscle structure and contractile function, which highlights the importance of this receptor for these processes through modulation of key genes such as *Myog*, *Myh3,* and *Cdkn1c* (Zammit et al. 2006; Osborn et al. 2011), as well as *Tnni2* (Marston and Zamora 2020), respectively.

The mechanisms by which ADRβ2 regulates myoblast differentiation were investigated by measuring changes in miRNA expression in differentiating myoblasts lacking ADRβ2 or exposed to beta 2 adrenergic antagonist or adenylyl cyclase activator. Our *in silico* analysis showed that *Adrβ2* was a potential target of miR‐374b‐5p. The finding that *Gsk3β* is upregulated in differentiating myoblasts from β2KO mice (Koike et al. 2022) and that miR‐374b‐5p, which targets *Gsk3β*, was downregulated in β2KO myoblasts at 24‐48 h of differentiation (Fig. 2), prompted us to validate the *Gsk3β* as a functional target of miR‐374b‐5p in myoblasts. Our data showed that ADRβ2 influenced *Gsk3β* expression during myoblast differentiation through a mechanism involving miRNA374b‐5p, whose expression was reduced. In addition, we found that the overexpression of miRNA374b‐5p rescued the differentiation defect of myoblasts lacking ADRβ2. Consistent with these findings, a study showed that ADRβ2 stimulation upregulated miR‐374b‐5p in C2C12 myotubes, resulting in the downregulation of the transcription factor forkhead box O1 (FoxO1) via ADRβ2‐cAMP signaling (Shimamoto et al. 2022). In contrast, it was previously described that miR‐374b decreased the mRNA and protein expression of Myf6 and inhibited C2C12 myoblast differentiation (Ma et al. 2015). We found that miR‐374b‐5p was downregulated in primary myoblasts from β2KO mice at 48 h of differentiation. The discrepancy between these studies may be due to differences in the type of muscle cells and duration of differentiation.

Considering that miR‐326‐3p regulates the differentiation of neural stem cells, lymphocytes, and adipocytes (Tang et al. 2009; Choi et al. 2016; Po et al. 2017; Corral‐Fernández et al. 2017), we investigated whether β2KO altered the expression of miR‐326‐3p in differentiating myoblasts. Our results demonstrated that β2KO downregulated miR‐326‐3p in myoblasts cultured for 24 h in PM and 24 or 48 h in DM. Furthermore, miR‐326‐3p was downregulated in myoblasts incubated with a beta 2 adrenergic antagonist for 48 h. In addition, overexpression of miRNA‐326‐3p rescued differentiation impairment of myoblasts lacking ADRβ2.

We noticed that the adenylyl cyclase activator attenuated the decrease in the fusion index of differentiating myoblasts lacking ADRβ2 accompanied by the rescue of miR374b and miR326 expression in these cells, suggesting that the canonical β2‐adrenergic pathway Gα‐adenylyl‐cyclase‐cAMP is involved in the modulation of ADRβ2‐dependent myoblast differentiation mediated by these miRs. In addition, our RNAseq data detected decreased expression of *Nr4a1* at 48 h of differentiation in β2KO myoblasts. Considering that *Nr4a1* is described as a cAMP response element‐binding protein (CREB) target gene (Kanzleiter et al. 2009; Maruoka et al. 2020), this result confirms that the absence of ADRβ2 in myoblasts affects the downstream signaling of the canonical β2‐adrenergic pathway Gα‐adenylyl‐cyclase‐cAMP. Collectively, these data reinforce the idea that the canonical β2‐adrenergic signaling modulates myoblast differentiation via miR‐374b and miR‐326.

Our in silico analysis showed that the 3′‐UTR of *Tnfrsf11a*, which encodes the RANK receptor, had a potential binding site for miR‐326‐3p. It is known that the RANK‐RANKL‐OPG axis regulates bone remodeling by controlling calcium homeostasis and osteoclastogenesis (Gostage et al. 2024). This axis is also described to control endothelial function, angiogenesis, cell proliferation, inflammation, immunity, tumorigenesis, metastasis, and brain signaling (Gostage et al. 2024). It is known that RANKL binds and activates RANK, and this interaction is inhibited by OPG, a soluble RANKL decoy receptor (Gostage et al. 2024). In addition, vesicular RANK, secreted by maturing osteoclasts, binds to osteoblastic RANKL and promotes bone formation by triggering RANKL reverse signaling, which activates Runt‐related transcription factor 2 (Ikebuchi et al. 2018).

In the skeletal muscle, it was reported that the overexpression of RANKL or the absence of OPG leads to muscle atrophy (Marcadet et al. 2022). Although mouse full‐length OPG‐Fc is demonstrated to improve muscle regeneration (Bouredji et al. 2021), little is known about the involvement of RANK‐RANKL‐OPG in different phases of muscle regeneration, including myogenic differentiation. Thus, here we validated *Tnfrsf11a* as a target for miRNA 326‐3p by dual‐luciferase reporter assays.

It is possible to suggest that the interaction between miRNA‐326‐3p and *Adrβ2* is mediated by the downregulation of *Tnfrsf11a* since we found that β2KO upregulated *Tnfrsf11a*, which is a target of miR‐326‐3p, with a consequent decrease in *Tnfrsf11* and *Rela* expression and NF‐kB activity during the terminal differentiation of myoblasts. Consistent with these findings, a study confirmed the effect of miR‐326 on NF‐κB by demonstrating that circ‐UQCRC2 knockdown in LPS‐treated MRC‐5 cells inhibited NF‐κB signaling by regulating the miR‐326/PDCD4 axis (Zhou et al. 2021).

A study showed that treatment with TNF‐α inhibited myogenesis by activating NF‐κB in C2C12 myoblasts. Further, this treatment decreased muscle protein expression in differentiating myoblasts but not in differentiated myotubes, demonstrating that TNF‐α selectively affected myogenesis at the myoblast stage (Langen et al. 2001). Our data showed that β2KO was associated with decreased NF‐κB activity in the terminal myogenic differentiation stage, downregulation of *Tnfrsf11*, and upregulation of *Tnfrsf11a*. These data suggest that ADRβ2 regulates terminal myogenic differentiation in a RANKL‐ and NF‐kB‐dependent manner, in which a possible involved mechanism could be a reverse signaling, where RANKL would stimulate terminal differentiation.

## 5 Conclusion

In conclusion, our results indicate that the β2 adrenergic signaling modulates myoblast differentiation through miR‐374b‐5p‐GSK3β and miR‐326‐3p/Tnfrsf11a axes. This study provides further insights into the molecular mechanisms involved in myogenic differentiation and may help develop effective strategies for skeletal muscle tissue repair and regeneration and treatment of muscle diseases.

## Author Contributions

Conceptualization: Tatiana E. Koike, Cesar S. Fuziwara, Elen H. Miyabara Experimental performance and/or data analysis: Tatiana E. Koike, Cesar S. Fuziwara, Audrei R. Santos, Fernanda V. B. Delpupo, Tábata L. Nascimento, Andrei Rozanski, Marcelo G. Pereira, Patrícia C. Brum, Edna T. Kimura, Thomas A. Rando, Elen H. Miyabara Funding acquisition: Elen H. Miyabara Project administration: Tatiana E. Koike, Cesar S. Fuziwara, Elen H. Miyabara Supervision: Elen H. Miyabara Manuscript preparation: Tatiana E. Koike, Cesar S. Fuziwara, Andrei Rozanski, Elen H. Miyabara Manuscript review: Tatiana E. Koike, Cesar S. Fuziwara, Audrei R. Santos, Fernanda V. B. Delpupo, Tábata L. Nascimento, Andrei Rozanski, Marcelo G. Pereira, Patrícia C. Brum, Edna T. Kimura, Thomas A. Rando, Elen H. Miyabara.

## Ethics Statement

The animal study protocols were approved by the Animal Research Ethics Committee of the Institute of Biomedical Sciences of the University of São Paulo (Protocol No. 106/2017, 7549191022).

## Conflicts of Interest

The authors declare no conflicts of interest.

## Uncited reference

Adrenoceptor regulation of the mechanistic target of rapamycin in muscle and adipose tissue.

## Supporting information


Supporting File

## Data Availability

The RNA‐seq data used in this publication were deposited in the National Center for Biotechnology Information and are accessible through SRA data: PRJNA1532947. Other data and figures are included in the article and supplementary data. Additional data will be made available upon reasonable request to Elen H. Miyabara.
