# Supplementary material for: β2 Adrenoceptor Signaling Modulates Myoblast Differentiation via miR‐374b‐5p/GSK3β and miR‐326‐3p/Tnfrsf11a Axes
Source: Cell Biol Int. 2026 Sep 26;50(10):e70214. doi: 10.1002/cbin.70214 (PMC13615415; doi:10.1002/cbin.70214)
Supplement: Supplementary file 1 [file CBIN-50-0-s001.docx]

**Supplemental Table S1.** Primer sequences for RT-qPCR.

| *GSK3b* | *GTC CGA CTG CGG TAT TTC TTC* | *CTC GAT GGC AGA TTC CAA AGG* |
| --- | --- | --- |
| *Tnni2* | *AGAGTGTGATGCTCCAGATAGC* | *AGCAACGTCGATCTTCGCA* |
| *Cdkn1c* | *AGAGAACTGCGCAGGAGAAC* | *TCTGGCCGTTAGCCTCTAAA* |
| *Tnfrsf11 (RANKL)* | *CAGCATCGCTCTGTTCCTGTA* | *CTGCGTTTTCATGGAGTCTCA* |
| *Tnfrsf11a (RANK)* | *GGACGGTGTTGCAGCAGAT* | *GCAGTCTGAGTTCCAGTGGTA* |
| *Tnfrsf11b (OPG)* | *ACCCAGAAACTGGTCATCAGC* | *CTGCAATACACACACTCATCACT* |
| *Rela (p65 NF-κB)* | *GAGGCACGAGGCTCCTTTTCT* | *GTAGCTGCATGGAGACTCGAACA* |
| *Myog* | *CGA TCT CCG CTA CAG AGG C* | *GTT GGG ACC GAA CTC CAG T* |
| *Myh3* | *CATCCGCATCCATTTTGGCAC* | *CTTGGTCGTAATCAGCAGCAG* |
| *Nr4a1* | *TTGAGTTCGGCAAGCCTACC* | *GTGTACCCGTCCATGAAGGTG* |
| *Taf1* | *TTAGGCCTGGAAAGGTGTTACG* | *CCTCCAAACAGATGGAACATTCT* |

**Supplemental Table S2.** RNA integrity number (RIN) and sequencing depth (million reads) of differentiating myoblasts (48 h in DM; differentiation medium) from WT and β2KO groups.

| **Samples** | **RIN** | **Sequencing depth**  (million reads) |
| --- | --- | --- |
| WT 1 | 9.4 | 12.1 |
| WT 2 | 9.9 | 10.9 |
| WT 3 | 9.7 | 12 |
| β2KO 1 | 9.8 | 11.4 |
| β2KO 2 | 9.7 | 12.6 |
| β2KO 3 | 9.9 | 11 |

**Supplemental Table S3.** Oligonucleotides for miRNA target validation using pmiR-Glo.

| **Oligonucleotide** | **5’- 3’ sequence** |
| --- | --- |
| *Gsk3β-*WT forward | TCGAGTAGCGGCCGCTAGTAACAAAATTTATTAT**TATTATA**TT |
| *Gsk3β-*WT reverse | CTAGAA**TATAATA**ATAATAAATTTTGTTACTAGCGGCCGCTAC |
| *Gsk3β-*Mut forward | TCGAGTAGCGGCCGCTAGTAACAAAATTTATTAT**TA***AATAT*TT |
| *Gsk3β-*Mut reverse | CTAGAA*ATATT***TA**ATAATAAATTTTGTTACTAGCGGCCGCTAC |
| *Tnfsrf11a-*WT forward | TCGAGTAGCGGCCGCTAGTAACACCATTTCCCGA**CCCAGAG**AT |
| *Tnfsrf11a-*WT reverse | CTAGAT**CTCTGGG**TCGGGAAATGGTGTTACTAGCGGCCGCTAC |
| *Tnfsrf11a-*Mut forward | TCGAGTAGCGGCCGCTAGTAACACCATTTCCCGA**CC***GTCTC*AT |
| *Tnfsrf11a-*Mut reverse | CTAGA**T***GAGAC***GG**TCGGGAAATGGTGTTACTAGCGGCCGCTAC |

**Supplemental File 1**

**Mouse Gsk3β 3' UTR Sequence**

```
1     acagcccccaggagccagctgcgcgggaaagaccagcacttacttgagtgccactcagca
61    acactggtcacgtttggaaagaaaattaaaaagaggaaaacaaaaacaaaaacaaaaaaa
121   ccctgttcattttagtgttcaattttttttattgttgttcttatttaaccttgtaaaata
181   tctatataaatacaaaccagtttcattgtattctcacttttcaggagatccaagaggtgg
241   ggagggttgggcggggaggagcagagcattaaacacaatctctcccacgacaatcttttc
301   ttttctttttaaagtcagaagtccgctattgtataccttaaaaaccagactcctgcctca
361   cgcccccaccgcaacagaagaaaacctgttctgtgctggtttattttctggatttgtttt
421   cttttaaagcctggcgtaagactttgatacagtgcacagcttgaaattggttgggagctt
481   agcaggtgtaactcaccagggacttactgtcacttgtacaatgtgtacttatccttagga
541   tttgaagacttgcctttggcgtgtgatgtcaggtatgacagacaaaaaaaaaaaagacaa
601   agaaaaaccaaggtgtattattggtagcccagggaggtcactaaaatctgaagctctaag
661   ggaagatcagagatggattacttaaggtttgttttgctcttgaccagtgcaggtgttgca
721   gatgccagcttaggaaggctgctgccaaaggagagagcgcgacagagtctgttgggccct
781   gaggtagatcttggttcacctgaccaaaggccgcctgcttctcccggtattgttttgagc
841   tggtaccctaggatgaggctggagatggtgttaagggggcagctctgggtgcatcggaaa
901   gcgaagaacaggaaggtggcaggccaggggcagatctcaccactgtgagtgagcttgtcc
961   agattttaataaagtcgtttcccttagaaaaacacttgagagaagacatcatagctaagt
1021  aatgtgaactgttagccagcctactgctttgttttccttactttaattgaaaaccaagct
1081  tgcaagaattgccacattcttatgcatagctctaattttaaatccaaatctagactctgt
1141  atttaatttctgcttttttaacatcatgctttttaaatttatttattgatgcatgtcaga
1201  tcggccactatgattttaagtggtagtaatattaaaaaccacacatcaaaataataaagt
1261  cacttgtatctgctgactttataggaaaactgattatattaatgtgtatatatgttttat
1321  atatacatatattcaatactgccttttctttttgtctacagtatcaaaattgactgactg
1381  aatcatgaaaagaatgttcccccaccatcaccattaagagttttatttttgttttctttg
1441  tttatcaatgaatggtgtaagaatcagtctcttgttttttgaagaaaaagcaatattcct
1501  tggaaagcaaggtggattgaaggattacgtttgtgtgaggaacagaactcataactagtt
1561  tgttgatacttttaaggttggtgtctttgtgggccttatatactctaaaatgaaccttgg
1621  tgcttatgggccattacttgacctatgaatctttaaggcacaatcagttatcttttacat
1681  ttaaagatcacttgagtaatggccaccgctccttccttccttcccactccctccccacac
1741  atctgctaaggtgagctgatgactagggctgtagggcccttggttatgtatgagacacag
1801  actcctttaccctcattacctgaccttaagtcacgttgaggtcttgccctccaggtggtt
1861  aggaagaggagtctcttggcggcccttatacaggccctagcaccctgccttgcttctccg
1921  accgtgtgtgtaactctccaagagagttgtattgtctgccgaaatgagttgatgctagaa
1981  agacatcagtgaggctgtgcggctggctgatctcttcaggccaccgaaggggtttctcag
2041  acctccgtttagatcagacttgacaagaaaggaagcaagcccaaagcaaacaaaggcgct
2101  tttggtaaagcgcttctcagataattgccgtgcgaaagacttctgaaattaagttatttg
2161  accgcatagttccttgaagggggtgacttggaagaccattttcagaaagaatttgtaaag
2221  aatctgagcaagcaggtctgtggcctgagaagggaacctgctttcagattaaactgccca
2281  gcccgacacagacagtactccagacttggtgtgcgtgctagagtccataaaagtcagtgc
2341  ttgttagtgaatactccccacccccagatacggggtaagagaagagtggaggaaagtcag
2401  gtgcttttctctgtgtgtgtgtgtgtgcgcgcgcgcgcgcgtgtgtgtgtgtgtgtgtgt
2461  gtgtgtgtgtgtgtgcgcgtgtgcgtgtgtgcgcacatgtgtgcgtccgtgtgtgtgtga
2521  gtgtgtgcctgtgtgtgtgcgcgcgcgcgtgtgtgtgtgtgtgtatgtgtgtgtgtatgt
2581  atgtgggcatgcatgcatatatgagggcttaggtgcacgatttttctttctcaaggatca
2641  tggcaggatcccagaactcttatccaagtgagatccaggtctctgaatatctttttgtaa
2701  ataataattaaaagctcctcaccaaattcaagcttgtatattatattttctttcaatgtt
2761  tttaaatttaagttttattgttttgtatgtaaatatgtggacccaggaactgttattaat
2821  gagcaaaaagttactgttcagggcagtgattctgttcaataaccagacaaaaatgtagac
2881  gagctttttaaagccatatagttttaactctgtacagtaggtaccggcctgtattattgt
2941  aacaataactctagcaatgtatagtgtatctatatagtttggagtgccttcgcttccatg
3001  tttttgggtttttttgttttgtttttgttttctgacttcctttatccatgtgtccctgtg
3061  gccccctttccctctgagtaacagcagaacagcacctttgcccactccacagtttgaatg
3121  gggagaggaaaccatgttggcagtctcacttcattctagccttgcctttgtttttggttc
3181  tgggtgtgtctgtgtcctcagtaacagtgaccggtttcattgcggatgcactcagtccgt
3241  ccgggacttggtggagcgctgggggccccagagctgaagggtgtcagatggcatgggttt
3301  gttcatcgcctctccaagccctggagtggatcctaagcagattctgggagctcccgagct
3361  tgctctctccttacctacttccctcccaaatgagagaaagattgggtttgttttattttt
3421  aaaaaaaaaaaaaatcactgtcttggtggcatttgggtgcccccgtgacattgttagcag
3481  ttactgcaggggtgctgtgcagatgagggctgcgagtggactctgagcccgttcctccct
3541  cctaccatcttgctagatgaaatatcattttaatttctttttttaaaaatcagtttaact
3601  gtgcctaatatggcccatttgaaagaaaaaaaaaagtattcgttttgccaccaactactt
3661  catgtgtcttgaatagaagaagaaaacaaaaaaaattcactaccaaaccaaaggttgcta
3721  tttttgagacatcacgagttcattgctcacggcagaagaatgcaccttctctcccatgac
3781  agactgtcagtttttaagtcttttttttttttttttttttttttttttttggcccatttc
3841  ttaggcttgtgttttagcaaagtatacctgcgtggccatcttgtccacgccaatgcagag
3901  gtcctaaaaggactccctctattctctatccctgtggacgtaaagacactggcatctctg
3961  ttaccttctcttccctttgcaagggttttaactttggaatcttccagagaaagtttggcc
4021  aatgccagatctcccagagttgggatttttgttattttttttctttgttttaaatcaaat
4081  ttgtgtctggttcctatttacactactgattgttctaatcccagagatgaatatgtttct
4141  cctctgtatgagaagtaagcaccctttatggtgaaacctgtctcttgtggtagaaacgga
4201  agccgctgaaaacaaacatcagcacaggagagccccaggcagaggtctcccttgagattc
4261  ttccgaagcaatcgttggggtctgggtcagtttcacagggttatgccattttattcaagt
4321  ccgttgctccgttgtgcagtctccaccatataagcaaaaatgacagccagtacataaggg
4381  gttagcttgacaaagtagacttccttgtgttcatttttaaggttttttttttcttaacta
4441  tatctgtctacaggcagatacagataggtatatggaaatgtgcttgcctgtaaaatttgc
4501  atttataaatgtgttgcgatggatcacttcggcctgtacacatccaattagcgtgaccac
4561  ttccatcttaaaaacaaatctaaacaaaatttattattattatatatatataaaggactg
4621  tgggttgtatacaaactattgcaaacacttgtgcaaatctgtcttgatataaaggaaaag
4681  caaacgctgtgtaacattactacttgaatgcctttgtgactgaattttttttttcatttt
4741  aaatataaacttttttgtggaaagtatgctcaatgtttttttccccctcccccccattcc
4801  cttgtaaatacattttgttctatgtgacttggtttggaaatagttaactggtactgtaat
4861  ttgcattaaataaaaagtaggttagcctggaaatgaaattaaacttcaccagtgtgtggt
4921  ctttatttcagtgcccatccccctctcttcacccttcccactctgccatcgccacatgca
4981  gtcacactgccgtctccactcctctgacagaggaaacatgaatccttgagagaaaaatca
5041  ggactctccttctctgagatgaccttgctctgatgtgtagacaggaggtgcagcttccag
5101  cttggaaaccccctggacttaatgtagagaaagctttgcagacactgctttggggaaaaa
5161  caaacaaaaaaccctgaaagggacagttgtaaaacatgtataagctgctgtctttggtta
5221  ctgtgtccatggtttggtgtggctgtatttattttaacttccatcttattagtaatggtc
5281  gttgggagtctttgtaaaccatgaacaccatggacattgggtcagtcccacccatgtaac
5341  caacacgtgtgtgagtctgctcatttccaatactggataatgtatgttaacatgttatgt
5401  ctcatagtgcaaacagaaacaccattttttagggctggctcattgtcaggcctaaaggtt
5461  agatataaggtcatgtgactgctgcttcaataaagacaaatttatattgg

Green miR-374b-5p site in miRDB
Blue miR-374b-5p site in TargetScan
```

**Supplemental Figure 1**

```
a

Gsk3β Wt  miR-374b-5p

      5’-AACAAAAUUUAUUATUAUUAUAU-3’   Gsk3b 3’-UTR
                        ||||||||
       3’-GUGAAUCGUCCAACAUAAUAUA-5’   miR-374b-5p

Gsk3β Mut miR-374b-5p

      5’-AACAAAAUUUAUUAUUAAAUAUU-3’   Gsk3b 3’-UTR
                        ||*****|
      3’- GUGAAUCGUCCAACAUAAUAUA-5’   miR-374b-5p
```

**b**

**pmiRGlo *Gsk3β*-Wt**

**XhoI	NotI	*Gsk3b* 3’UTR Wt	XbaI**

```
5’-  TCGAGTAGCGGCCGCTAGTAACAAAATTTATTATTATTATATT       -3’
         |||||||||||||||||||||||||||||||||||||||
3’-      CATCGCCGGCGATCATTGTTTTAAATAATAATAATATAAGATC   -5’
```

**pmiRGlo *Gsk3β*-Mut**

**XhoI	NotI	*Gsk3b* 3’UTR Mut	XbaI**

```
5’-  TCGAGTAGCGGCCGCTAGTAACAAAATTTATTATTAAATATTT       -3’
         |||||||||||||||||||||||||||||||||||||||
3’-      CATCGCCGGCGATCATTGTTTTAAATAATAATTTATAAAGATC   -5’
```

**Fig. S1. (a)** Potential interaction between miR-374b-5p and *Gsk3β* 3'-UTR based on the analysis of the miRDB database. The miR-374b-5p seed region is shown in bold, and mutations are shown in asterisks. **(b)** The 3'-UTR region was cloned into the *Xho*I and *Xba*I sites of the pmiRGlo luciferase reporter vector. Primers are shown in **Supplemental Table S3.**

**Supplemental Figure 2**

```
a

Tnfrsf11a WT miR-326-3p

5’     AACACCAUUUCCCGACCCAGAGA - 3’
5’                    |||||||
3        UGACCUCCUUCCCGGGUCUCC   miR-326-3p

Tnfrsf11a Mut miR-326-3p

5’     AACACCAUUUCCCGACCGUCUCA - 3’
5’                    ||*****
3        UGACCUCCUUCCCGGGUCUCC   miR-326-3p
```

**b**

**pmiRGlo *Tnfrsf11a*-Wt**

**XhoI	NotI	*Tnfrsf11a* 3’UTR Wt	XbaI**

```
5’-  TCGAGTAGCGGCCGCTAGTAACACCATTTCCCGACCCAGAGAT      -3’
3’-      CATCGCCGGCGATCATTGTGGTAAAGGGCTGGGTCTCTAGATC  -5’
```

**pmiRGlo *Tnfrsf11a*-Mut**

**XhoI	NotI	*Tnfrsf11a* 3’UTR Mut	XbaI**

```
5’-  TCGAGTAGCGGCCGCTAGTAACACCATTTCCCGACCGTCTCAT      -3’
3’-      CATCGCCGGCGATCATTGTGGTAAAGGGCTGGCAGAGTAGATC  -5’
```

**Fig. S2. (a)** Potential interaction between miR-326-3p and *Tnfrsf11a* 3'-UTR based on the analysis of the TargetScan database. The miR-326-3p seed region is shown in bold, and mutations are shown in asterisks. **(b)** The 3'-UTR region was cloned into the *Xho*I and *Xba*I sites of pmiRGlo. Primers are shown in **Supplemental Table S3.**

**Supplemental Figure 3**

**Fig. S3.** Expression of a) miR-374b-5p and b) miR-326-3p in uninjured control and cryoinjured tibialis anterior (TA) muscles at 10 days post-injury (dpi) from β2KO and WT mice. Data are expressed as mean and SEM (n= 3-5 mice per group). ANOVA followed by Tukey's test. *p ≤ 0.05. Groups: Control: uninjured muscles; Injured: cryoinjured TA muscles analyzed at 10 dpi; WT: wild type; β2KO: ADRβ2 knockout mice.

**Supplemental Figure 4**

**Fig. S4.** a) Representative immunostaining of Myh3-positive WT myoblasts incubated with the ADRβ2 antagonist ICI in differentiation media (DM) for 48 h. Blue: DAPI (nuclear staining) and Red: Myh3. Bar: 50 μm. Fusion index (%) of WT myoblasts treated with ICI in DM for 48 h. Groups: Control (treated with the vehicle PBS); ICI (treated with 20 μM ICI-118, 551). Data are expressed as mean ± SEM; **p* ≤ 0.05 vs. the Control group (T student test), n = 3 biological replicates per group. Expression of b) miR-374b-5p (n= 3 biological replicates), and c) miR-326-3p (n= 3-4 biological replicates) in WT myoblasts incubated with ICI in DM for 48 h. Groups: Control (treated with the vehicle PBS); ICI (treated with 20 μM ICI-118, 551). (T student test) Data are expressed as mean ± SEM; **p* ≤ 0.05 vs. Control group. d) Representative immunostaining of desmin-positive β2KO myoblasts incubated with the adenylyl cyclase activator forskolin (FK) in DM for 48 h. Blue: DAPI (nuclear staining) and Red: desmin. Bar: 50 μm. Fusion index (%) of β2KO myoblasts treated with FK in DM for 48 h. Groups: WT Control (WT myoblasts treated with the vehicle DMSO); β2KO Control (β2KO myoblasts treated with the vehicle DMSO); and β2KO FK (β2KO myoblasts treated with 40 μM FK). Data are expressed as mean ± SEM; *p* ≤ 0.05 versus WT Control (ANOVA followed by Tukey's test), n = 3-4 biological replicates per group. Expression of e) miR-374b-5p (n= 4-5 biological replicates), and f) miR-326-3p (n= 3-5 biological replicates) in β2KO myoblasts incubated with FK in DM for 48 h. Groups: WT Control (WT myoblasts treated with the vehicle DMSO); β2KO Control (β2KO myoblasts treated with the vehicle DMSO); and β2KO FK (β2KO myoblasts treated with 40 μM FK). (ANOVA followed by Tukey's test) Data are expressed as mean ± SEM; **p* ≤ 0.05 vs. WT Control group.
